# Clusters of Coronavirus Disease in Communities, Japan, January–April 2020

**DOI:** 10.3201/eid2609.202272

**Published:** 2020-09

**Authors:** Yuki Furuse, Eiichiro Sando, Naho Tsuchiya, Reiko Miyahara, Ikkoh Yasuda, Yura K. Ko, Mayuko Saito, Konosuke Morimoto, Takeaki Imamura, Yugo Shobugawa, Shohei Nagata, Kazuaki Jindai, Tadatsugu Imamura, Tomimasa Sunagawa, Motoi Suzuki, Hiroshi Nishiura, Hitoshi Oshitani

**Affiliations:** Kyoto University, Kyoto, Japan (Y. Furuse, K. Jindai);; Nagasaki University, Nagasaki, Japan (E. Sando, I. Yasuda, K. Morimoto);; Tohoku University, Sendai, Japan (N. Tsuchiya, Y.K. Ko, M. Saito, T. Imamura, S. Nagata, H. Oshitani);; National Center for Global Health and Medicine, Tokyo, Japan (R. Miyahara);; Niigata University, Niigata, Japan (Y. Shobugawa);; Japan International Cooperation Agency, Tokyo (T. Imamura);; National Institute of Infectious Diseases, Tokyo (T. Sunagawa, M. Suzuki);; Hokkaido University, Sapporo, Japan (H. Nishiura)

**Keywords:** respiratory infections, severe acute respiratory syndrome coronavirus 2, SARS-CoV-2, SARS, COVID-19, 2019 novel coronavirus disease, zoonoses, viruses, coronavirus, epidemiology, transmission, Japan

## Abstract

We analyzed 3,184 cases of coronavirus disease in Japan and identified 61 case-clusters in healthcare and other care facilities, restaurants and bars, workplaces, and music events. We also identified 22 probable primary case-patients for the clusters; most were 20–39 years of age and presymptomatic or asymptomatic at virus transmission.

Coronavirus disease (COVID-19) typically causes febrile illness with respiratory symptoms (*1*,*2*), and many countries worldwide have been affected. Before characterizing COVID-19 as a pandemic in March 2020 (*3*), the World Health Organization advised countries to take measures to reduce spread of the virus, including identifying cases and clusters, isolating patients, tracing contacts, and preventing community transmission (*4*). Several countries have reported on the characteristics of a small number of clusters of COVID-19 cases (*5*,*6*). However, few comprehensive reports provide an overview of clusters of COVID-19 cases in communities and the significance of such clusters. We analyzed 61 COVID-19 clusters among various communities in Japan and identified 22 probable primary cases that might have contributed to the disease incidence in clusters.

## The Study

**Figure 1:**
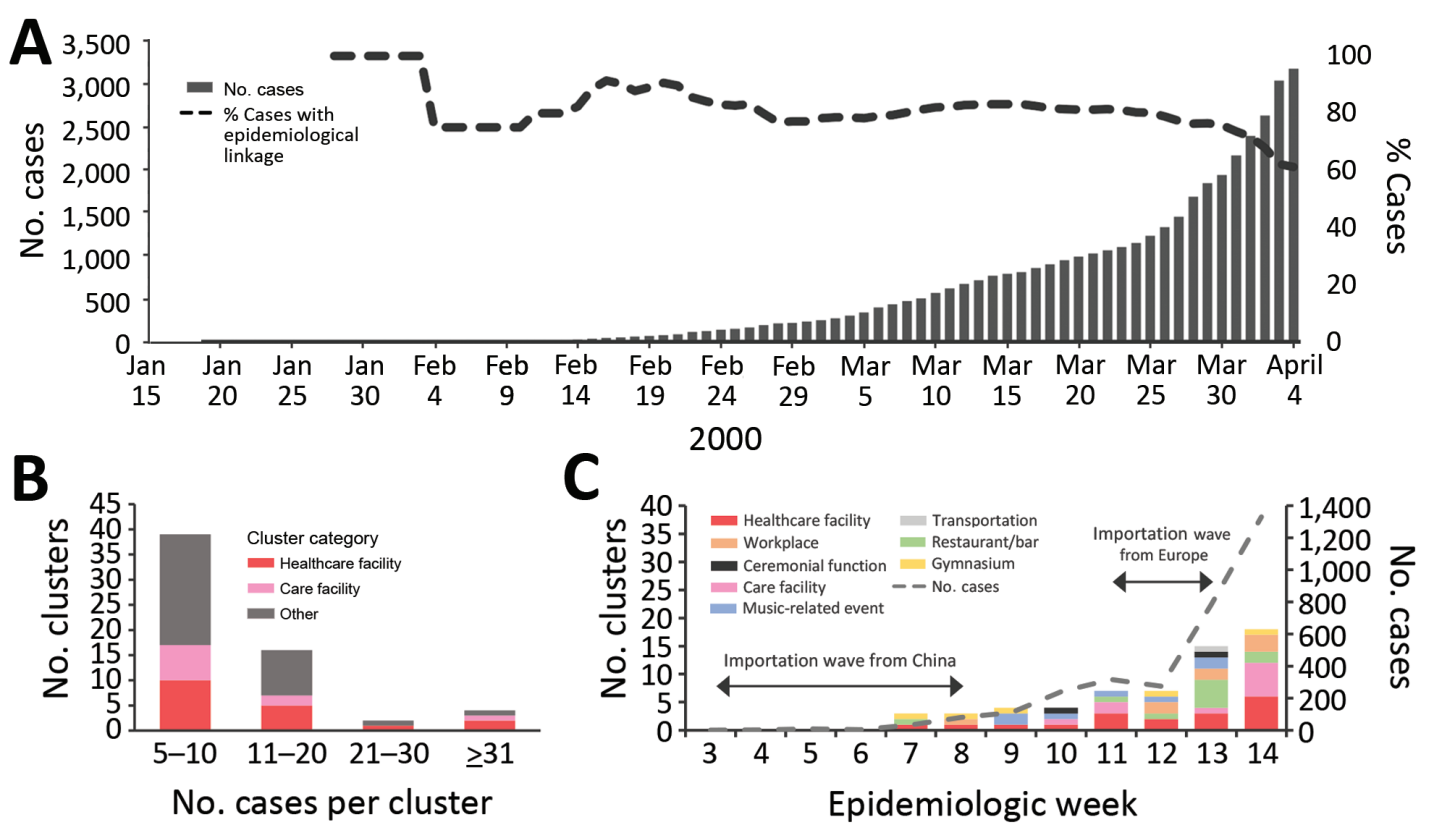
Analysis of 61 clusters of coronavirus disease (COVID-19) cases in communities in Japan, January 15–April 4, 2020. A) Cumulative number of COVID-19 cases, including the proportion of local cases with epidemiologic links to known confirmed cases. B) Distribution of clusters by number of cases in a cluster by category. C) Incidence of clusters of cases according to epidemiologic week as determined by date of confirmation of the first case in a cluster. Incidence of COVID-19 cases (weekly number of newly reported cases) in Japan and timing of two importation waves are also displayed. Epidemiologic week 3 corresponds to January 15, 2020, in panel A. The data and trend of imported cases were previously reported and described by Furuse et al. (*8*).

We analyzed COVID-19 cases in Japan reported during January 15–April 4, 2020. All COVID-19 cases confirmed by reverse transcription-PCR in Japan must be reported to the Ministry of Health, Labour and Welfare. Through case interviews, local health authorities collected demographic and epidemiologic information, such as possible source of infection and contact and travel history. During the study period, a total of 3,184 laboratory-confirmed COVID-19 cases, including 309 imported cases, were reported. Among cases of local transmission, 61% (1,760/2,875) had epidemiologic links to known cases (Figure 1, panel A). We excluded 712 cases detected on a cruise that was anchored at Yokohama Port, Japan, from February 3 through March 1 (*7*).

We defined a cluster as >5 cases with primary exposure reported at a common event or venue, excluding within-household transmissions. Our definition also excluded cases with epidemiologic links to secondary transmission. For example, in the following scenario we would exclude cases A and B: boy A is a friend of boy B whose grandmother C contracted nosocomial COVID-19 in a nursing home from which ≥5 cases were reported; although all 3 have symptoms develop and are diagnosed with COVID-19, we would consider only grandmother C part of a cluster from the nursing home.

By investigating the epidemiologic links among cases, we identified 61 COVID-19 clusters in various communities. We observed clusters of COVID-19 cases from 18 (30%) healthcare facilities; 10 (16%) care facilities of other types, such as nursing homes and day care centers; 10 (16%) restaurants or bars; 8 (13%) workplaces; 7 (11%) music-related events, such as live music concerts, chorus group rehearsals, and karaoke parties; 5 (8%) gymnasiums; 2 (3%) ceremonial functions; and 1 (2%) transportation-related incident in an airplane. Most (39/61; 64%) clusters involved 5–10 cases (Figure 1, panel B). The largest cluster involved >100 cases in a hospital, including nosocomial infections and staff infections. The largest non–healthcare-related cluster we observed was among >30 persons who attended a live music concert, including performers, audience members, and event staff. Healthcare and care facilities accounted for >50% of clusters at epidemiologic weeks 11 and 14 (Figure 1, panel C).

**Figure 2:**
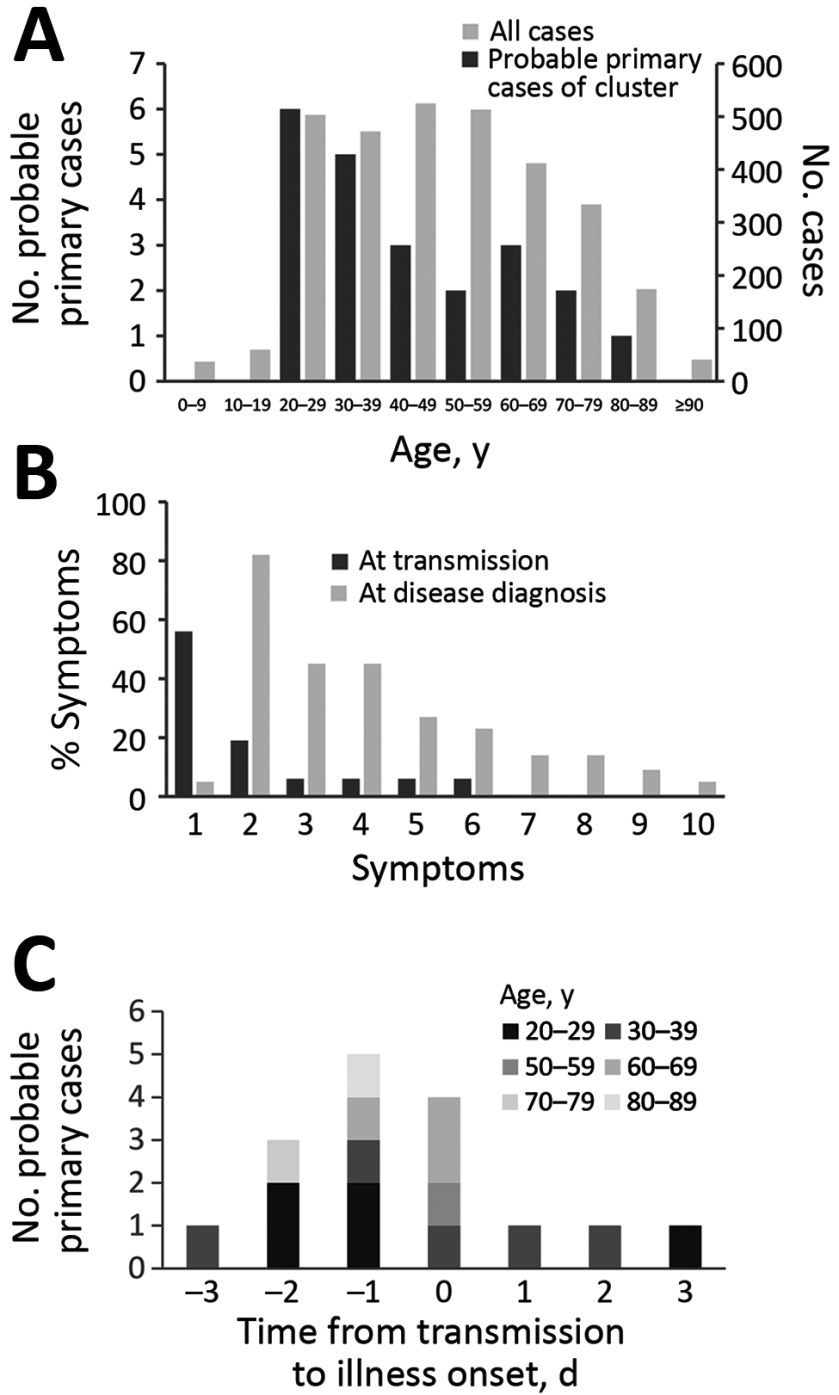
Analysis of probable primary cases of coronavirus disease (COVID-19) among 22 clusters in communities, Japan. A) Age ranges of probable primary COVID-19 cases in clusters. Age distribution among all COVID-19 cases in Japan is provided as reference. B) Proportions of symptoms among probable primary cases of COVID-19 clusters at transmission (n = 16) and among at laboratory confirmed diagnosis (n = 22). 1, Asymptomatic; 2, fever; 3, fatigue; 4, cough; 5, sore throat; 6, headache; 7, arthralgia or myalgia; 8, runny nose; 9, diarrhea; 10, difficulty breathing. C) Distribution of probable primary cases of COVID-19 clusters by time of transmission compared with illness onset by age groups (n = 16). Six cases were excluded because the time of transmission was undetermined.

We identified 22 probable primary case-patients who had symptoms develop before they had contact with other case-patients in a cluster or who had prior epidemiologic links before contact with a cluster. We did not identify probable primary cases for nosocomial clusters. We believe these 22 case-patients contributed to the incidence of clusters. Demographic data show that 9 (41%) probable primary case-patients were female and 13 (59%) were male. The most frequently observed age groups among probable primary cases were 20–29 years (n = 6; 27%) and 30–39 years (n = 5, 23%) (Figure 2, panel A). For 16 clusters, we determined the date of transmission from probable primary case-patients to other case-patients in a cluster and found 41% (9/22) of probable primary case-patients were presymptomatic or asymptomatic at the time of transmission; only 1 had a cough at the time of transmission (Figure 2, panel B). Of the 22 probable primary case-patients, 45% (10/22) had cough at the time of diagnosis. Of the 16 probable primary case-patients with the determined date of transmission, transmission occurred one day before illness onset for 5 (31%) case-patients and on the same day of illness onset for 4 (25%) case-patients (Figure 2, panel C). All age groups demonstrated presymptomatic or asymptomatic transmission.

## Conclusions

We investigated clusters of COVID-19 cases and probable primary cases in Japan during January 15–April 4, 2020. We found that healthcare facilities, such as hospitals, and care facilities, such as nursing homes, were the primary sources of clusters, some of which had >100 cases. Japan experienced 2 waves of imported COVID-19 cases, after which local transmission occurred and the epidemic grew (*8*). Of note, clusters of COVID-19 cases at healthcare and care facilities predominated at epidemiologic weeks 11 (March 9–15) and 14 (March 30–April 4), which corresponds to ≈3 weeks after the 2 waves of imported cases (Figure 1, panel C). Healthcare and care facilities might be located at the end of the local transmission chain because clusters in those facilities only became evident several weeks after community transmission persisted.

We noted many COVID-19 clusters were associated with heavy breathing in close proximity, such as singing at karaoke parties, cheering at clubs, having conversations in bars, and exercising in gymnasiums. Other studies have noted such activities can facilitate clusters of infection (*9*,*10*). Japan’s Prime Minister’s Office and the Ministry of Health, Labour and Welfare announced 3 situations that could increase the risk for COVID-19 cases and advised the population to avoid the “Three Cs”: closed spaces with poor ventilation, crowded places, and close-contact settings (*11*).

Among the probable primary COVID-19 cases we identified from non-nosocomial clusters, half (11/22) were 20–39 years of age, which is younger than the age distribution of all COVID-19 cases in Japan (Figure 2, panel A). We do not know whether social, biological, or both factors play a role in the difference in transmission patterns between the younger and older persons. We also noted probable primary COVID-19 case-patients appear to transmit the virus and generate clusters even in the absence of apparent respiratory symptoms, such as cough.

Our study has some limitations. The epidemiologic investigation mostly relied on voluntary cooperation. Because some case-patients could not disclose contact history, epidemiologic links and clusters of cases might have missed. Recall bias is likely because Japan did not use digital devices for contact tracing and information was obtained only through interviews. In addition, we could not calculate a secondary attack rate from probable primary cases because data were unavailable for denominator, such as the number of persons present in the places where clusters of cases were detected.

Active case finding and investigation are key to establishing links to other cases or transmission events. Detecting clusters of cases can lead to effective quarantine of close contacts and to the identification of risk factors for the formation of such clusters (*12*). Our findings provide further information and insight on clusters of COVID-19 cases in communities that can aid in the ongoing efforts to curb the global pandemic.
